# Supplementary material for: Observation of interlayer plasmon polaron in graphene/WS2 heterostructures
Source: Nat Commun. 2024 May 8;15:3845. doi: 10.1038/s41467-024-48186-4 (PMC11519396; doi:10.1038/s41467-024-48186-4)
Supplement: Supplementary file 1 — Supporting Information [file 41467_2024_48186_MOESM1_ESM.pdf]

Supplementary Information

Observation of interlayer plasmon polaron in graphene/WS_2 heterostructures

Søren Ulstrup*,[1] Yann in 't Veld,[2] Jill A. Miwa,[1] Alfred J. H. Jones,[1] Kathleen M. McCreary,[3] Jeremy T. Robinson,[3] Berend T. Jonker,[3] Simranjeet Singh,[4] Roland J. Koch,[5] Eli Rotenberg,[5] Aaron Bostwick,[5] Chris Jozwiak,[5] Malte Rösner*,[2] and Jyoti Katoch*[4]

[1]*Department of Physics and Astronomy, Interdisciplinary Nanoscience Center, Aarhus University, 8000 Aarhus C, Denmark*

[2]*Institute for Molecules and Materials, Radboud University, 6525 AJ Nijmegen, the Netherlands*

[3]*Naval Research laboratory, Washington, D.C. 20375, USA*

[4]*Department of Physics, Carnegie Mellon University, Pittsburgh, Pennsylvania 15213, USA*

[5]*Advanced Light Source, E. O. Lawrence Berkeley National Laboratory, Berkeley, California 94720, USA*

* *Email: ulstrup@phys.au.dk, m.roesner@science.ru.nl, jkatoch@andrew.cmu.edu*

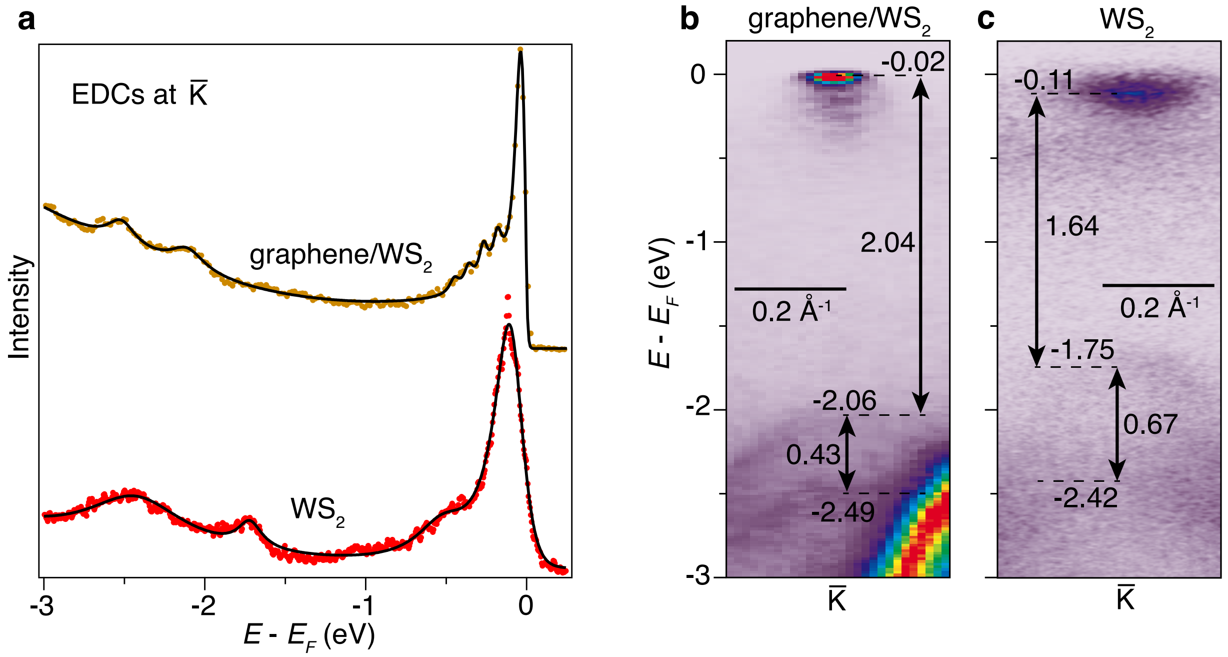

FIG. S1. Extraction of band gaps from ARPES. **a,** Energy distribution curves (EDCs) at $\bar{\mathrm{K}}$ in the heavily doped situations shown in Figs. 1(c) and 1(e) of the main paper. The black curves represent fits to a modelled spectral function with several Lorentzian peaks on a linear background and including a Fermi-Dirac cut-off. **b-c,** ARPES spectra around $\bar{\mathrm{K}}$, displaying the VBM and CBM of SL WS_2 (b) with and (c) without a graphene overlayer. VBM and CBM peak positions obtained from the EDC analysis in (a) and the resulting energy differences are stated in units of eV. The error bars are ± 0.02 eV.

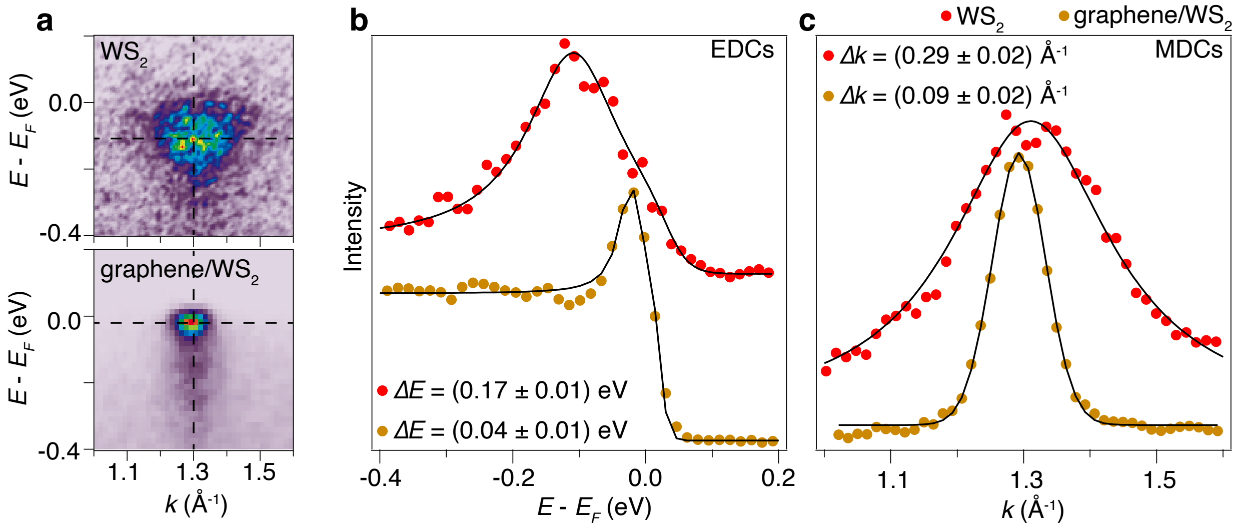

FIG. S2. ARPES linewidths of conduction band states. **a,** ARPES spectra in the conduction band region of WS_2 and graphene/WS_2, corresponding to the data shown in Figs. 1(d) and 1(f) of the main paper. **b,** EDCs (markers) extracted along the vertical dashed lines in (a) with fits (black curves) to a single Lorentzian peak multiplied by a Fermi-Dirac function. The background is approximated by a constant offset. The linewidth of the Lorentzian peak is stated as ΔE. **c,** Momentum distribution curves (MDCs) extracted at the EDC peak energies marked by horizontal dashed lines in (a) and fits to a Lorentzian peak convoluted with a Gaussian. The resulting momentum linewidth is stated as Δk.

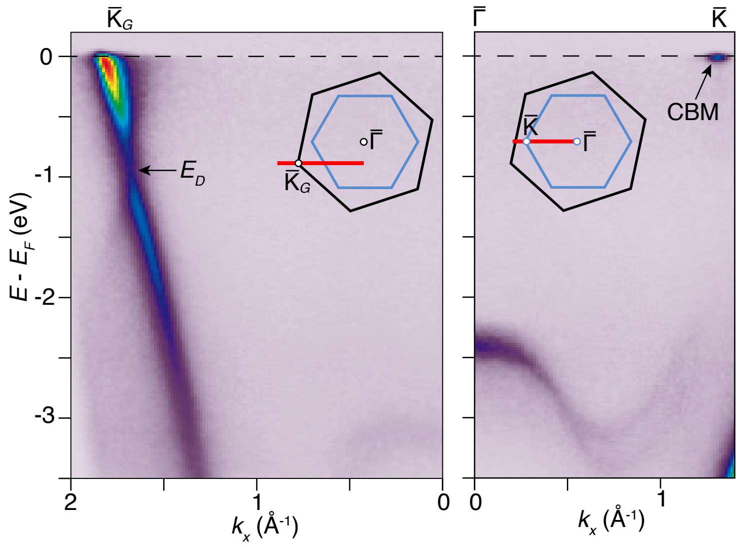

FIG. S3. ARPES data for doped graphene/WS_2. The black and blue Brillouin zones (BZs) correspond to graphene and WS_2, respectively. The rotation between the BZs corresponds to a twist angle of $(18.1 \pm 0.3)^\circ$ between graphene and WS_2. The red line demarcates the direction of the ARPES cut. The graphene carrier concentration is $(5.2 \pm 0.1) \cdot 10^{13}$ cm^{-2}. The Dirac energy E_D and WS_2 CBM are indicated by arrows.

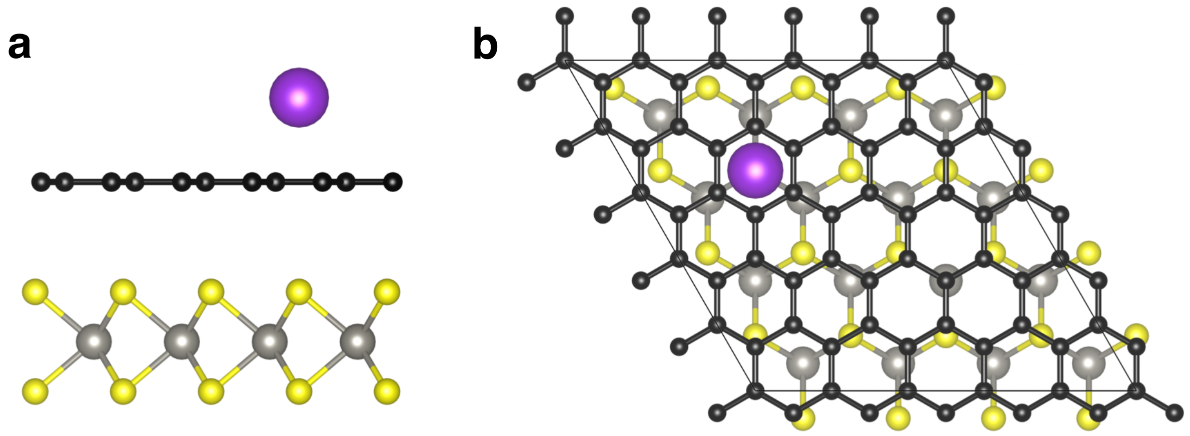

FIG. S4. DFT heterostructure model. **a-b,** View from the (a) side and (b) top of the utilized 4×4 WS_2 / 5×5 graphene supercell with K doping.

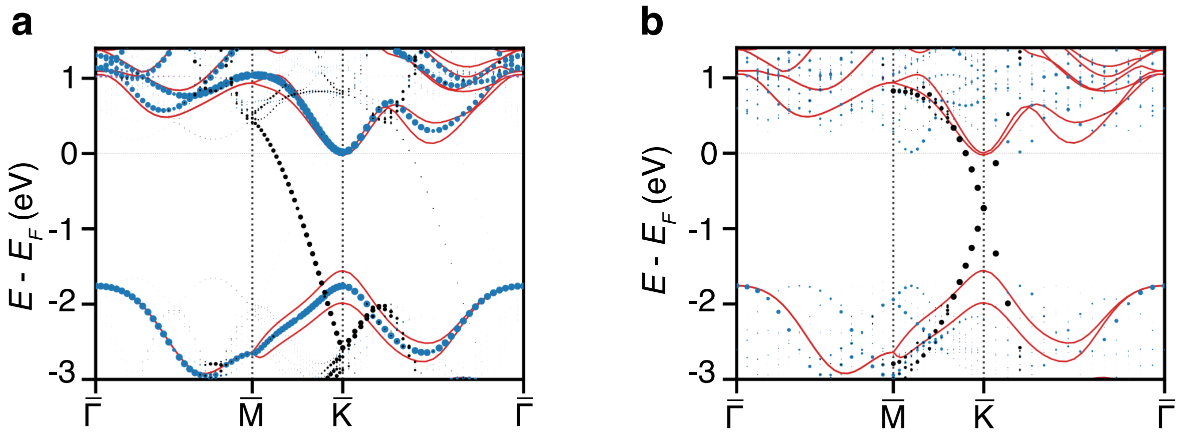

FIG. S5. DFT electronic structure. **a-b,** Band structure of WS_2 with SOC (red line) plotted together with the unfolded band structure without SOC (dots) in the (a) WS_2 and (b) graphene primitive BZs. Blue and black dots represent W and C weights.
